# Assessment of Serum Minerals in Subclinical Hypothyroid and Overt Hypothyroid Patients

**DOI:** 10.7759/cureus.16944

**Published:** 2021-08-06

**Authors:** Ram K Jat, Anil K Panwar, Pradeep Agarwal, Chandrashekhar Sharma, Dharam P Bansal, Arpit Pareek, Ambika Tyagi, Monali Mathur

**Affiliations:** 1 General Medicine, Mahatma Gandhi Medical College and Hospital, Jaipur, IND; 2 General Medicine, Sawai Man Singh Medical College and Hospital, Jaipur, IND

**Keywords:** calcium, phosphorus, subclinical hypothyroidism, thyroid stimulating hormone, thyroid hormones

## Abstract

Background

Hypothyroidism, the commonest form of hormonal dysfunction, is due to thyroid hormone deficiency or its impaired activity. Homeostasis of the metabolism of minerals is regulated by thyroid hormones. If there is any disorder of the thyroid it will lead to disturbances of metabolism of minerals.

Aim

To study and compare serum calcium and serum phosphorus levels in patients of subclinical hypothyroidism and correlation of these parameters with thyroid-stimulating hormone (TSH) levels.

Materials and methods

This study included 70 patients with subclinical hypothyroidism, 70 patients with overt hypothyroidism, and 70 age- and sex-matched healthy controls. Thyroid profile (estimation of free triiodothyronine [FT3], free thyroxine [FT4], TSH) was done. In both cases and controls serum calcium and serum phosphorus levels were estimated.

Results

Serum calcium and phosphorus levels in patients of subclinical hypothyroidism was 8.75 ± 0.40 mg/dL and 3.80 ± 0.62 mg/dL, respectively. In patients with hypothyroidism it was 8.37 ± 0.52 mg/dL and 4.10 ± 0.75 mg/dL, respectively, and in controls it was 9.67 ± 0.97 mg/dL and 3.70 ± 0.71 mg/dL, respectively. Difference between these groups was statistically significant (p<0.05 ). Mean serum calcium and phosphorus for patients with TSH level <10 was 8.81 ± 0.33 mg/dL and 3.67 ± 0.60 mg/dL, respectively, and for TSH level >10 was 8.59 ± 0.51 mg/dL and 4.12 ± 0.54 mg/dL, respectively. The difference between both groups was statistically significant (p<0.05) for calcium, phosphorus .

Conclusions

In subclinical hypothyroidism serum calcium and serum phosphorus levels are significantly altered. Regular follow-up and estimating serum levels of these minerals in subclinical hypothyroidism patients should be done so it is beneficial to give mineral supplementations to prevent bone complications during the treatment of the disease.

## Introduction

Hypothyroidism is one of the most common endocrine disorders worldwide [1]. The prevalence of hypothyroid is 10-11% in India [2]. Hypothyroidism is caused by decreased levels of thyroid hormones and it is among the most common endocrine disorders. Subnormal activity of the thyroid gland in hypothyroidism leads to mental and physical slowing because of a decrease in the basal metabolic rate [3]. The prevalence of spontaneous hypothyroidism is between 1% and 2% and is more common in older women and 10 times more common in women than in men [4].

Subclinical hypothyroidism, defined as an elevated serum thyroid-stimulating hormones (TSH) level with normal levels of free thyroxine (FT4) affects up to 10% of the adult population [5]. Subclinical (without obvious symptoms) hypothyroidism (low thyroid function) describes a situation in which thyroid function is only mildly low, so that the blood level of thyroxine remains within the normal range but the blood level of TSH is elevated [6]. Subclinical hypothyroidism, which is defined as elevated thyroid-stimulating hormone (TSH) levels with free thyroxine concentrations within the reference range, is a common disorder that increases with age and affects up to 18% of the elderly, with a higher prevalence in women compared to men [7].

Thyroid hormones have a vital role in the growth and of the skeletal system and its maturation. TSH is a ligand hormone between the hypothalamic-pituitary axis and the thyroid gland. TSH has long been recognized to act on the thyroid gland to control follicle development and thyroid hormone production and secretion. Beyond the thyroid, TSH has also been shown to have additional effects on other tissues. TSH can exert a direct effect on bone metabolism independently of the peripheral thyroid hormone (thyroxine [T4] and triiodothyronine [T3]) levels [8]. The role of thyroid hormones in phosphorus and calcium balance in the body by direct action on bone turnover have variously been reported in the literature. Phosphorus and ionized calcium metabolism are frequently altered in thyroid disease. A negative calcium balance may ultimately result in hyperthyroid osteopenia [9]. Calcium and phosphorus are important parts of the metabolic pathways regulated by the thyroid hormones [10].

Our aim in this study is to assess serum calcium and serum phosphorus levels in newly diagnosed hypothyroidism, subclinical hypothyroidism, and euthyroidism and compare them.

## Materials and methods

A hospital-based observational study was conducted in the Department of Medicine, Mahatma Gandhi Medical College Hospital, Jaipur, in 2018-19. Total 210 subjects included in this study were divided into three groups - euthyroid, subclinical hypothyroid, and hypothyroid. Seventy subjects included in each group were selected according to the inclusion and exclusion criteria and serum calcium, phosphorus levels were measured. Subjects aged 18-70 years were included in this study. Patients with a history of preexisting hepatic disease, renal disease, bone diseases, and other major medical conditions, patients with a history of alcohol abuse, diabetes mellitus, pediatric age group and persons on mineral supplementation or any medications that might affect serum calcium, magnesium, and phosphorous concentration were excluded from this study. In statistical methods, the p-value is obtained by applying a one-way analysis of variance (ANOVA).

## Results

**Figure 1:**
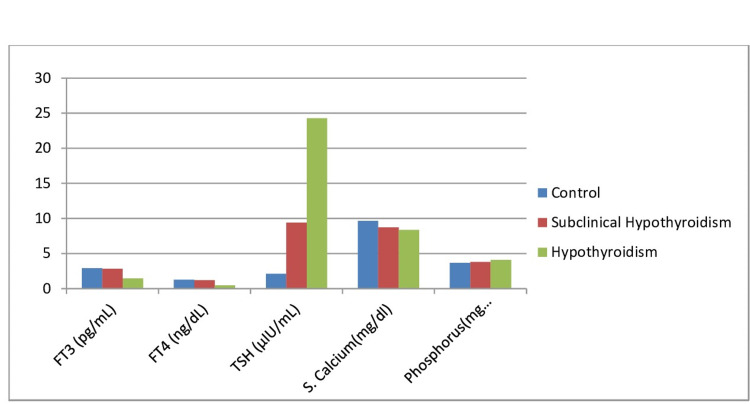
Comparison of FT3, FT4, TSH, Serum Calcium and Phosphorous in Cases and Controls FT3: free triiodothyronine; FT4: free thyroxine; TSH: thyroid-stimulating hormone

Mean FT3, FT4, and TSH in patients of subclinical hypothyroidism was 2.83 ± 0.44 pg/mL, 1.22 ± 0.36 ng/dL, and 9.41 ± 4.10 µIU/mL, respectively. In patients with hypothyroidism it was 1.48 ± 0.41 pg/mL, 0.49 ± 0.14 ng/dL, and 24.29 ± 20.69 µIU/mL, respectively, And in controls it was 2.94 ± 0.48 pg/mL, 1.26 ± 0.45 ng/dL, and 2.12 ± 1.20 µIU/mL, respectively. Difference in FT3, FT4, and TSH was statistically significant(p<0.001 ) in all three groups. Serum calcium and phosphorus levels in patients of subclinical hypothyroidism was 8.75 ± 0.40 mg/dL and 3.80 ± 0.62 mg/dL, respectively. In patients with hypothyroidism they were 8.37 ± 0.52 mg/dL and 4.10 ± 0.75 mg/dL, respectively, and in controls it was 9.67 ± 0.97 mg/dL and 3.70 ± 0.71 mg/dL, respectively. Difference between these groups was statistically significant (p<0.05 ) (Table 1, Figure 1).

**Table 1:** Comparison of Age, T3, T4, TSH, Serum Calcium, and Phosphorous Controls and Cases *p-value as obtained on applying one-way analysis of variance T3: triiodothyronine; T4: thyroxine; TSH: thyroid-stimulating hormone

Parameters	Control	Subclinical Hypothyroidism	Hypothyroidism	p-value*
FT3 (pg/mL)	2.94 ± 0.48	2.83 ± 0.44	1.48 ± 0.41	<0.001
FT4 (ng/dL)	1.26 ± 0.45	1.22 ± 0.36	0.49 ± 0.14	<0.001
TSH (µIU/mL)	2.12 ± 1.20	9.41 ± 4.10	24.29 ± 20.69	<0.001
S. Calcium (mg/dl)	9.67 ± 0.97	8.75 ± 0.40	8.37 ± 0.52	<0.001
Phosphorus (mg/dl)	3.70 ± 0.71	3.80 ± 0.62	4.10 ± 0.75	0.002

**Figure 2:**
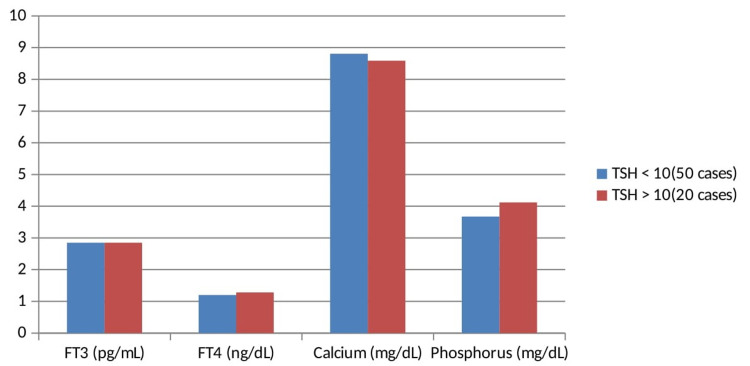
Comparison of FT4 Levels in Sub-Clinical Hypothyroid Group Divided on the Basis of TSH Levels FT3: free triiodothyronine; FT4: free thyroxine; TSH: thyroid-stimulating hormone

In this study out of 70 cases of subclinical hypothyroidism 50 cases had TSH level <10 while 20 cases had TSH level >10 and the mean for these patients was 7.38 ± 1.35 µIU/mL and 14.49 ± 4.28 µIU/mL, respectively (Table 2, Figure 2).

**Table 2:** Comparison of FT4 Levels in Sub-Clinical Hypothyroid Group Divided on the Basis of TSH Levels FT3: free triiodothyronine; FT4: free thyroxine; TSH: thyroid-stimulating hormone

Parameter	TSH <10 (50 cases)	TSH >10 (20 cases)	p-value
FT3 (pg/mL)	2.85 ± 0.42	2.85 ± 0.42	0.669
FT4 (ng/dL)	1.20 ± 0.34	1.28 ± 0.40	0.401
Calcium (mg/dL)	8.81 ± 0.33	8.59 ± 0.51	0.036
Phosphorus (mg/dL)	3.67 ± 0.60	4.12 ± 0.54	0.005

Mean FT3 and FT4 for patients with TSH level <10 was 2.85 ± 0.42 pg/mL and 1.20 ± 0.34 ng/mL, respectively, and for TSH level >10 it was 2.80 ± 0.49 pg/mL and 1.28 ± 0.40 ng/mL, respectively. This difference was statistically non-significant (p>0.05).

Mean serum calcium and phosphorus for patients with TSH level <10 was 8.81 ± 0.33 mg/dL and 3.67 ± 0.60 mg/dL, respectively and for TSH level >10 it was 8.59 ± 0.51 mg/dL, 4.12 ± 0.54 mg/dL, respectively. Both groups had statistically significant difference (p<0.05).

In this study, serum calcium had a significant negative correlation and serum phosphorus levels had a significant positive correlation with TSH levels (p<0.05) (Table 3).

**Table 3:** Correlation of Serum Calcium, Phosphorous with TSH Among Cases TSH: thyroid-stimulating hormone

Parameters	Correlation coefficient (r)	p-value
TSH vs S. Calcium	-0.243	0.003
TSH vs S. Phosphorus	0.317	<0.0001

## Discussion

The mean age in our study in patients with subclinical hypothyroidism was 40.4 ± 13.33 years, in patients with hypothyroidism was 41.07 ± 10.78 years, and in controls was 37.30 ± 13.19. It shows age-matched cases and controls (p=0.165 ).

In this study, we found mean serum calcium levels in patients of subclinical hypothyroidism, hypothyroidism, and in controls was 8.75 ± 0.40 mg/dL, 8.37 ± 0.52 mg/dL and 9.67 ± 0.97 mg/dL respectively. These results show significantly (p<0.001 ) low values of serum calcium in patients of subclinical hypothyroidism and overt hypothyroidism in comparison to euthyroid patients.

Our results correlate with a study conducted by Kavitha MM et al [11], Arvind Bharti et al [12] - they found the mean serum calcium levels were significantly (p<0.001) low in subclinical hypothyroidism patients and overt hypothyroidism patients in comparison to euthyroid patients. According to Zahra N et al study, the Level of calcium and phosphorus were determined in patients with hypothyroidism. The level of calcium decreased in female hypothyroid patients (3.1263 ± 0.56122) as compared to control (8.7714 ± 0.23604) but the level of phosphorus (8.3316 ± 0.35037) in female patients with hypothyroidism was increased than the control (3.3143 ± 0.49473). The same trends were observed in the males when calcium and phosphorus levels were determined in hypothyroidism patients. Results were statistically significant at p<0.01 [13]. Long-term hyperthyroidism resulted in net negative calcium ion (Ca2+) balance in response to increased skeletal turnover. It is noteworthy here that a long-term hyperthyroid state is also associated with malabsorption of Ca2+ and increased bone resorption. On the other hand, hyperthyroidism is known to increase renal blood flow, glomerular filtration rate (GFR) and reabsorption of inorganic phosphate (Pi), Ca2+, and sodium ion (Na+) [14].

In our study, we found mean serum phosphorus level in patients of subclinical hypothyroidism, hypothyroidism, and in controls was 3.80 ± 0.62 mg/dL, 4.10 ± 0.75 mg/dL, and 3.70 ± 0.71 mg/dL, respectively. These results show significantly (p<0.001 ) high values of serum phosphorus levels in patients of subclinical hypothyroidism and overt hypothyroidism in comparison to euthyroid patients. Our results correlate with the study conducted by Kavitha MM et al [11] and Arvind Bharti et al [12] - they found the mean serum phosphorus levels were significantly (p<0.001)high in subclinical hypothyroidism patients and overt hypothyroidism patients in comparison to euthyroid patients. The mechanisms of thyroid hormone-induced bone resorption include cyclic adenosine monophosphate (cAMP)-mediated, increased sensitivity of beta-adrenergic receptors to catecholamines, increased sensitivity of bone cells to parathyroid hormone (PTH), osteoclast activator factor, and interleukin-1 (IL-1)-mediated increased bone resorption. Previous studies revealed that hypocalcemia is seen in hypothyroidism this is mainly due to the low levels of PTH and low levels of calcitriol [15].

We divided the patients of subclinical hypothyroidism into two subgroups according to the level of TSH. We found 50 cases of TSH level <10 and while 20 cases of TSH level >10 and mean TSH levels for these patients was 7.38 ± 1.35 µIU/mL and 14.49 ± 4.28 µIU/mL, respectively.

In this study, we found that mean serum calcium for patients with TSH level <10 was 8.81 ± 0.33 mg/dL and for TSH level >10 was 8.59 ± 0.51 mg/dL. This shows significantly low levels of calcium in patients with TSH levels of >10 (p<0.05). We also found in this study significant (p-value=0.003) negative correlation (r= -0.243) between TSH levels and serum calcium levels. It shows as TSH levels increase, serum calcium levels decreases. Our results correlate with the study conducted by Kavitha MM et al [11 ] which found a significant (p<0.001) negative correlation between calcium and TSH levels. A contrast study conducted by Bharti et al [12] in 2015 showed a negative but nonsignificant (p=0.45) correlation between calcium and TSH levels.

In this study, we found that mean serum phosphorus for patients with TSH level <10 was 3.67 ± 0.60 mg/dL and for TSH level >10 it was 4.12 ± 0.54 mg/dL. This shows significantly high levels of phosphorus in patients with TSH levels of >10 (p<0.05). We also found in this study a significant (p=<0.001) positive correlation (r=0.317) between TSH levels and serum phosphorus levels. It shows as TSH levels increase, serum phosphorus levels increases. Our results correlate with the study conducted by Kavitha MM et al [11] and Murgod et al [16] - they found a significant (p<0.05 ) positive correlation between phosphorus and TSH levels. Another study conducted by Bharti et al [12] in 2015 showed a positive but nonsignificant(p=0.45) correlation between phosphorus and TSH levels. Another contrast study conducted by Gammage MD et al [17] in 1986 showed a negative correlation of phosphorus with TSH.

## Conclusions

To conclude, the present study gives us an idea that the derangement in level serum calcium and phosphorus is associated with skeletal deformities so it needs to be studied further. Considering all this we suggest that patients with hypothyroidism and subclinical hypothyroidism should be regularly checked for serum calcium and phosphorus. Early detection and treatment can prevent further complications and will be helpful during the management of thyroid patients.
